# The effects of traditional Chinese exercises on anxiety and depression in adults: a systematic review and network meta-analysis

**DOI:** 10.3389/fpubh.2025.1582923

**Published:** 2025-04-09

**Authors:** Huan Feng, YanJing Li, QingChuan Wang, Ye Tao, ZhiHua Wang

**Affiliations:** ^1^College of Physical Education, Sichuan University, Chengdu, China; ^2^College of Physical Education, Jiangxi Normal University, Nanchang, China; ^3^College of Sports, Chengdu Sport University, Chengdu, China

**Keywords:** Tai Chi, traditional Chinese exercises, TCEs, anxiety, depression, meta-analysis

## Abstract

**Objective:**

To systematically evaluate the comparative effectiveness of traditional Chinese exercises (TCEs) and other interventions for managing anxiety and depression in adults using a network meta-analysis approach.

**Design:**

Systematic review and network meta-analysis.

**Methods:**

Literature search was conducted in Embase, PubMed, Web of Science, and Cochrane Library from inception to December 31, 2024. Randomized controlled trials (RCTs) involving TCEs for adults with anxiety or depression were included. Two reviewers independently screened studies, extracted data, and assessed risk of bias using the ROB2 tool. Bayesian network meta-analyses were performed to compare the effectiveness of interventions. The surface under the cumulative ranking area (SUCRA) was used to rank the interventions.

**Data sources:**

Embase, PubMed, Web of Science, and Cochrane Library.

**Eligibility criteria for selecting studies:**

RCTs involving adults aged 18 years and older, intervention groups engaging in TCEs, comparator groups with no intervention, routine treatment, or distinct interventions, reported outcomes related to anxiety or depression assessed using validated tools, and studies published in English or Chinese.

**Results:**

A total of 82 RCTs were included, with 4,501 participants. For anxiety, Liu Zi Jue (SUCRA = 99.5%), Tai Chi (SUCRA = 87.6%), and CBT (SUCRA = 75.3%) were the most effective interventions. For depression, Tai Chi (SUCRA = 95.5%), Yijinjing (SUCRA = 89.2%), and CBT (SUCRA = 83.6%) ranked as the most effective. TCEs demonstrated comparable or superior efficacy to well-established interventions like CBT. The study also found that TCEs have high adherence rates, low costs, and the potential for large-scale promotion and value.

**Conclusion:**

This systematic review and network meta-analysis provides evidence for the efficacy of TCEs, particularly Tai Chi, Liu Zi Jue, and Yijinjing, in managing anxiety and depression in adults. Despite limitations such as heterogeneity in intervention protocols and study populations, the findings suggest that these exercises offer therapeutic benefits and may serve as accessible, cost-effective, and culturally-relevant treatment options. Further research is needed to establish optimal dosages, assess long-term effects, and evaluate generalizability across diverse contexts.

**Systematic review registration:**

CRD42025637146.

## 1 Introduction

Anxiety and depression are among the most prevalent mental health disorders worldwide, significantly impairing quality of life and imposing substantial burdens on global health systems. Anxiety disorders, as defined in the Diagnostic and Statistical Manual of Mental Disorders (DSM-5), encompass several conditions characterized by excessive, persistent fear and worry lasting at least six months (1). These include generalized anxiety disorder, panic disorder, social anxiety disorder, and specific phobias (2, 3). Depression, or major depressive disorder, is clinically defined by persistent feelings of sadness or emptiness, markedly diminished interest in activities (anhedonia), and associated cognitive-somatic changes present nearly every day for at least two weeks and significantly interfering with normal functioning (4, 5). The World Health Organization estimates that over 264 million people globally suffer from depression, a leading cause of disability (6), while anxiety disorders affect approximately 284 million individuals worldwide (7). Current treatments, though effective, often involve adverse side effects, high costs, and limited accessibility, with treatment coverage as low as 20% in low-income countries (8, 9).

Traditional Chinese exercises (TCEs), such as Tai Chi, Qigong, and Baduanjin, combine physical movements, meditation, and controlled breathing, offering potential benefits for mental health (10–12). Evidence suggests TCEs positively influence mental health outcomes by improving autonomic regulation, reducing stress, and fostering emotional well-being. Tai Chi has been shown to enhance mood regulation and reduce depressive symptoms across various populations, including older adults and cancer survivors (13–15). Similarly, Qigong interventions have demonstrated efficacy in mitigating anxiety and depressive symptoms across various clinical populations (16). Nevertheless, comprehensive evaluations comparing different TCEs’ relative effectiveness remain limited (17, 18). This presents an opportunity for research advancement, as various TCE modalities differ in their movement complexity, intensity, meditative components, and theoretical foundations, suggesting they may have differential effects on mental health outcomes that remain unexamined when studied in isolation. Additionally, existing meta-analyses often focus on individual interventions or specific populations, thereby limiting the generalizability of their conclusions. Without head-to-head comparisons of multiple TCEs within a unified analytical framework, it remains unclear whether certain practices offer superior therapeutic benefits for particular symptom profiles or are more suitable for implementation in different healthcare settings. Network meta-analysis (NMA) offers a methodological approach that addresses these considerations by simultaneously comparing multiple interventions and establishing their relative efficacy (19–25).

This study aims to systematically evaluate various TCEs’ effects on anxiety and depression in adults through NMA, incorporating evidence from diverse randomized controlled trials. By synthesizing evidence from high-quality studies, this analysis seeks to provide clinicians, researchers, and policymakers with enhanced understanding of the role of TCEs in mental health care and to inform the development of evidence-based guidelines.

## 2 Methods

This study adheres to the PRISMA 2020 (26) statement for reporting and has been prospectively registered on the PROSPERO (27) platform (International Prospective Register of Systematic Reviews and Meta-Analyses) with the registration ID: CRD42025637146.

### 2.1 Data sources and searches

The literature search was conducted in databases including Embase, PubMed, Web of Science, and Cochrane Library, from inception to December 31, 2024. Two investigators independently analyzed and screened studies using the following keywords: (“Traditional Chinese exercise” OR “Tai Chi” OR “Qigong” OR “Wu Qin Xi” OR “Baduanjin” OR “Yijinjing” OR “Liuzijue”) AND (“Anxiety*” OR “Hypervigilance” OR “Nervousness” OR “Angst” OR “Anxiousness”) AND (“Depression*” OR “Mood disorders” OR “Mental health intervention”) AND (“randomized controlled trial” OR “random*” OR “clinic*” OR “control” OR “trial”). Only studies published in Chinese and English were included.

### 2.2 Inclusion and exclusion criteria

The inclusion criteria for this review were as follows: studies involving adults aged 18 years and older; intervention groups engaging in TCEs such as Tai Chi, Qigong, Wu Qin Xi, and Baduanjin; Comparator groups comprised no intervention (blank control), standard care, or alternative exercise interventions distinct from the intervention group, including aerobic training, CBT, stretching exercises, and others. Included outcome measures were reported outcomes related to anxiety, depression, or both, assessed using validated tools; randomized controlled trials (RCTs); and studies published in English. The exclusion criteria included duplicate publications, animal experiments, protocol registrations, studies involving individuals with physical disabilities, and articles with missing data. For studies with missing data, authors were contacted via email, and if no response was received within 2 weeks, the study was excluded from the review.

### 2.3 Data extraction

Two reviewers (YJ and QC) independently screened and selected studies based on the predefined inclusion and exclusion criteria. The retrieved articles were imported into EndNote 21 for deduplication, screening, and data extraction. The following information was extracted from each study: (1) general details, including the study title, first author, and year of publication; (2) participant characteristics, such as mean age and sample size; (3) study characteristics, including study design, type of intervention in both experimental and control groups, and treatment duration; and (4) outcome data, including reported means and standard deviations (SD). For studies with missing data or incomplete reporting of results, the corresponding authors were contacted via email to request the necessary information.

### 2.4 Quality assessment

The quality of the included studies was evaluated using the Risk of Bias 2 (ROB2) (28) tool, recommended by the Cochrane Handbook for Systematic Reviews of Interventions. Two reviewers (YJ and QC) independently assessed the risk of bias across five domains: bias arising from the randomization process, bias due to deviations from intended interventions, bias due to missing outcome data, bias in the measurement of the outcome, and bias in the selection of the reported result. Each domain was rated as “low risk,” “some concerns,” or “high risk,” with an overall judgment for each study. Discrepancies were resolved through discussion with a third reviewer.

### 2.5 Statistical analysis

Statistical analyses were performed using STATA 18 software (StataCorp LLC, College Station, TX, USA) and the netmeta package in R software (R Foundation for Statistical Computing, Vienna, Austria). The standardized mean difference (SMD) with 95% confidence intervals (CIs) was used as the effect size measure. Heterogeneity among studies was assessed using the I^2^ statistic, which quantifies the proportion of variability due to heterogeneity rather than chance. The classification of I^2^ values was as follows: I^2^ < 25% indicates low heterogeneity, I^2^ 25–50% indicates moderate heterogeneity, and I^2^ > 50% indicates high heterogeneity. The effectiveness of interventions was ranked based on the surface under the cumulative ranking area (SUCRA) and cumulative probability plots, and funnel plots were utilized to evaluate potential publication bias. For interventions with varying doses that formed closed-loop structures, both local and global inconsistency tests were conducted to assess the agreement between direct and indirect comparisons. The global inconsistency test was performed using the design-by-treatment interaction test, while the local inconsistency test was conducted using the node-splitting method, where *p*-values were calculated to assess inconsistency. A *p*-value >0.05 in either test indicated that the results were consistent, suggesting no significant difference between direct and indirect evidence. Additionally, consistency was deemed satisfactory when the 95% CI of the inconsistency factor included zero. A consistency model was applied to analyze the data, rank the interventions, and determine the most effective approach.

## 3 Results

### 3.1 Study selection

**Figure 1:**
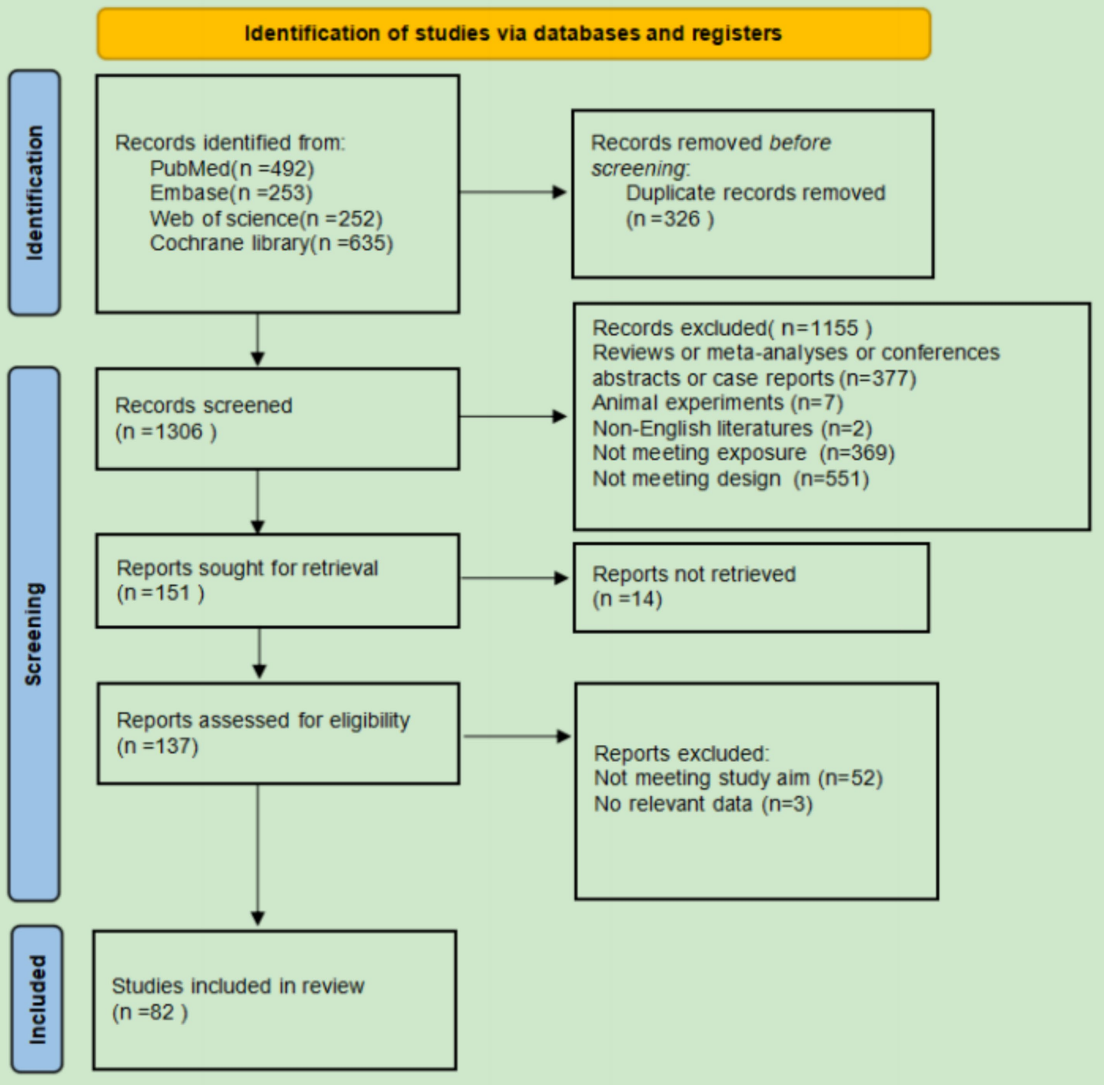
PRISMA literature search flow diagram.

A total of 1,632 records were identified from PubMed (*n* = 492), Embase (*n* = 253), Web of Science (*n* = 252), and Cochrane Library (*n* = 635). After removing duplicates (*n* = 326), 1,306 records were screened. 151 reports were sought for retrieval, and 137 reports were assessed for eligibility. Ultimately, 82 studies met the inclusion criteria. Records were excluded for being reviews, meta-analyses, conference abstracts, or case reports (*n* = 377), animal experiments (*n* = 7), non-English literature (*n* = 2), or not meeting exposure or design criteria (*n* = 920). The final selection process is shown in Figure 1.

### 3.2 Study characteristics

A total of 82 studies were included in the analysis, spanning multiple countries. China contributed the largest number of studies (46), followed by the United States (13), Australia (4), Spain and South Korea (3 each), Turkey (2), while Poland, Thailand, Portugal, Sweden, the United Kingdom, and Brunei each contributed 1 study. The age of participants varied from 24 to 76 years across studies, with specific age ranges reported for both the intervention and control groups. The duration of interventions ranged from 10 to 22 weeks. The sample sizes for the intervention groups and control groups ranged from 14 to 136 participants. Various intervention protocols were used, including Tai Chi, Baduanjin, Qigong, and others, while control protocols included home exercises, cognitive therapy, and waiting controls. Outcomes were assessed using multiple scales, including the Hamilton Anxiety and Depression Scale, the Self-Rating Anxiety Scale, the Self-Rating Depression Scale, the Beck Depression Inventory-II, the State–Trait Anxiety Inventory, the Geriatric Depression Scale, and others, evaluating different parameters such as anxiety, depression, and combined outcomes. Further details on the characteristics of the included studies are presented in Appendix.

### 3.3 Risk of bias assessment

**Figure 2:**
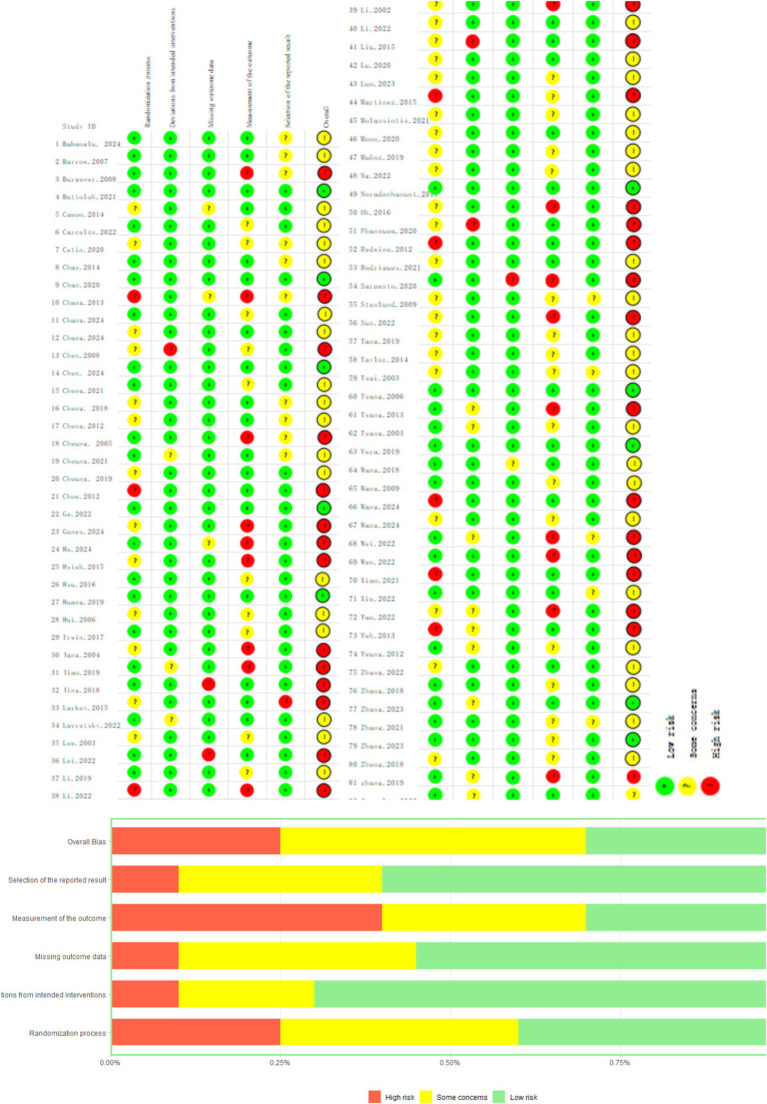
Risk of bias assessment of included studies.

The risk of bias was evaluated across five domains using the ROB2 tool. Overall, 35.3% of studies demonstrated a low risk of bias, 43.1% had some concerns, and 21.6% exhibited high risk. The highest bias was observed in the measurement of outcomes domain, where only 29.4% of studies showed low risk, while 33.3% had high risk and 37.3% presented some concerns. This elevated bias level is primarily due to the inherent difficulty in blinding participants and personnel in exercise intervention studies, as participants inevitably know whether they are performing specific exercises, and researchers must know group assignments to deliver appropriate interventions. In comparison, other domains showed better performance: randomization process (54.9% low risk), deviations from intended interventions (72.5% low risk), missing outcome data (70.6% low risk), and selection of reported results (58.8% low risk). The specific data can be found in Figure 2.

### 3.4 Results of network meta-analysis

**Figure 3:**
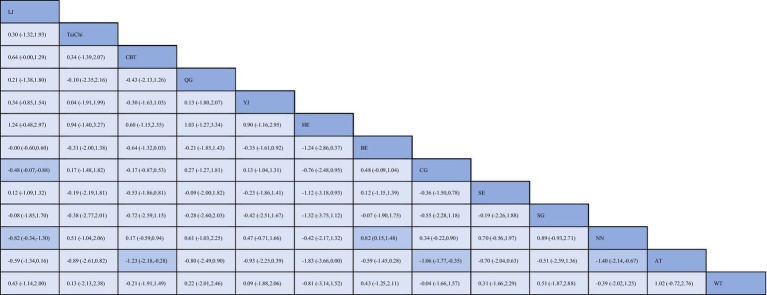
League table for network meta-analysis of anxiety. BE, Baduanjin; TC, Tai Chi; QG, Qigong; YJ, Yijinjing; SG, Sham Control; CBT, CBT; CG, Blank Control; HE, Home Exercise; NN, Standard Care; WT, Waiting Control; SE, Stretching; AT, Aerobic Training; LJ, Liu Zi Jue.

**Figure 4:**
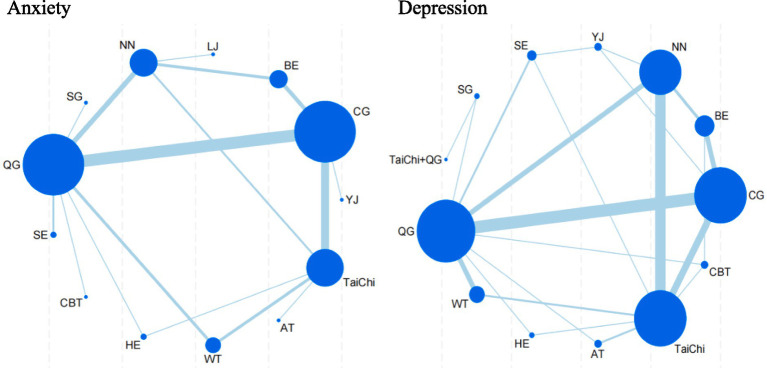
Network plot for anxiety and depression. BE, Baduanjin; TC, Tai Chi; QG, Qigong; YJ, Yijinjing; SG, Sham Control; CBT, CBT; CG, Blank Control; HE, Home Exercise; NN, Standard Care; WT, Waiting Control; SE, Stretching; AT, Aerobic Training.

**Figure 5:**
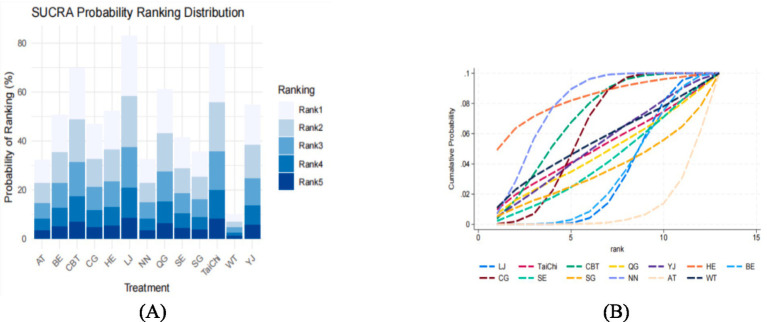
Cumulative probability ranking plot **(A)** and SUCRA ranking bar chart **(B)**.

Figure 3 summarizes the results of pairwise comparisons between different interventions and control measures for reducing anxiety. Yijinjing demonstrated significantly greater effectiveness in reducing anxiety scores compared to the blank control group (SMD = −0.48, 95% CI = −0.07 to −0.88, *p* < 0.05) and the health education group (SMD = −0.82, 95% CI = −0.34 to −1.30, *p* < 0.05). CBT also showed superior efficacy over aerobic training (SMD = −1.23, 95% CI = −2.18 to −0.28, *p* < 0.05), the blank control group (SMD = −1.06, 95% CI = −1.77 to −0.35, *p* < 0.05), and the health education group (SMD = −1.40, 95% CI = −2.14 to −0.67, *p* < 0.05). Additionally, Baduanjin demonstrated significantly greater efficacy than the health education group (SMD = 0.82, 95% CI = 0.15 to 1.48, *p* < 0.05). No statistically significant differences were observed in other pairwise comparisons (*p* > 0.05). Detailed results are presented in Figure 3. The SUCRA ranking analysis further evaluated the comparative effectiveness of TCEs and other interventions in reducing anxiety. Liu Zi Jue (SUCRA = 99.5%) emerged as the most effective intervention, followed by Tai Chi (SUCRA = 87.6%), CBT (SUCRA = 75.3%), Qigong (SUCRA = 69.8%), Yijinjing (S UCRA = 60.2%), Aerobic Training (SUCRA = 56.4%), Baduanjin (SUCRA = 43.7%), and Stretching (SUCRA = 39.6%). The cumulative probability plot corroborates these findings, with Liu Zi Jue consistently ranked as the most effective intervention overall. Tai Chi and CBT ranked second and third, respectively, both demonstrating substantial benefits in alleviating anxiety symptoms. In contrast, Baduanjin and Stretching ranked lower, indicating relatively limited efficacy compared to other interventions (Figure 4). These results highlight the robust therapeutic benefits of Liu Zi Jue, Tai Chi, and CBT in managing anxiety in adults. Their consistent performance across various measures provides compelling evidence for their integration into clinical practice and treatment strategies. Further details can be found in Figure 5.

**Figure 6:**
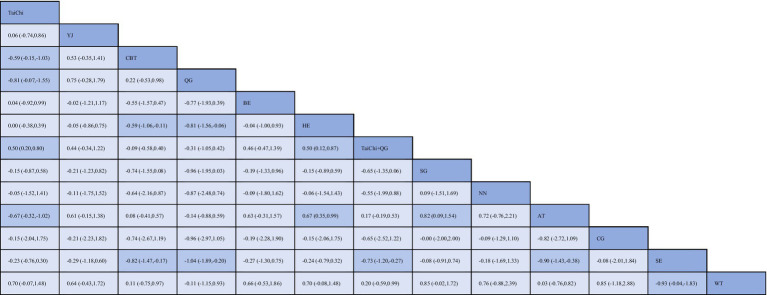
League table for network meta-analysis of depression. BE, Baduanjin; TC, Tai Chi; QG, Qigong; YJ, Yijinjing; SG, Sham Control; CBT, CBT; CG, Blank Control; HE, Home Exercise; NN, Standard Care; WT, Waiting Control; SE, Stretching; AT, Aerobic Training.

As presented in Figure 6, 14 pairwise comparisons among the depression interventions revealed significant differences (*p* < 0.05). Specifically, Tai Chi demonstrated significantly greater efficacy in improving depression scores compared to CBT (SMD = −0.59, 95% CI = −0.15 to −1.03, *p* < 0.05), Qigong (SMD = −0.81, 95% CI = −0.07 to −1.55, *p* < 0.05), and Aerobic Training (SMD = 0.67, 95% CI = −0.32 to −1.02, *p* < 0.05). Similarly, CBT showed superior performance over Home Exercise (SMD = −0.59, 95% CI = −1.06 to −0.11, *p* < 0.05) and Stretching (SMD = −0.82, 95% CI = −1.47 to −0.17, *p* < 0.05). In addition, Qigong significantly outperformed both Home Exercise (SMD = −0.81, 95% CI = −1.56 to −0.06, *p* < 0.05) and Stretching (SMD = −1.04, 95% CI = −1.89 to −0.20, *p* < 0.05). Tai Chi combined with Qigong demonstrated significantly greater improvements in depression scores compared to both Tai Chi alone (SMD = 0.50, 95% CI = 0.20 to 0.80, *p* < 0.05) and Home Exercise (SMD = 0.50, 95% CI = 0.12 to 0.87, *p* < 0.05). Furthermore, Aerobic Training exhibited significantly superior outcomes compared to Home Exercise (SMD = 0.67, 95% CI = 0.35 to 0.99, *p* < 0.05), Sham Control (SMD = 0.82, 95% CI = 0.09 to 1.54, *p* < 0.05), and Stretching (SMD = −0.90, 95% CI = −1.43 to −0.38, *p* < 0.05). Lastly, Stretching was found to be significantly more effective than Waiting Control (SMD = −0.93, 95% CI = −0.04 to −1.83, *p* < 0.05). No other pairwise comparisons showed statistically significant differences (*p* > 0.05).

**Figure 7:**
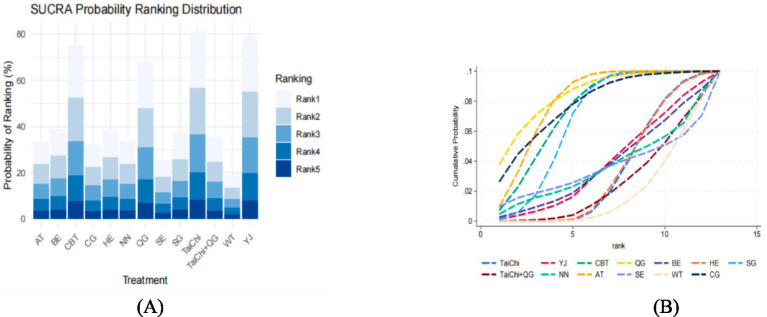
Cumulative probability ranking plot **(A)** and SUCRA ranking bar chart **(B)**.

The SUCRA analysis and the area under the cumulative probability curve further quantified the relative effectiveness of the interventions in alleviating depression. Among the evaluated treatments, Tai Chi (SUCRA = 95.5%) ranked as the most effective, followed by Yijinjing (SUCRA = 89.2%), CBT (SUCRA = 83.6%), Qigong (SUCRA = 75.3%), Home Exercise (SUCRA = 68.7%), Baduanjin (SUCRA = 62.4%), Stretching (SUCRA = 55.1%), and Tai Chi combined with Qigong (SUCRA = 48.9%). These findings are further illustrated in Figure 7, which highlights the dominant performance of Tai Chi, followed by Yijinjing and CBT, while other interventions demonstrated comparatively moderate or limited efficacy.

These results underscore the therapeutic potential of Tai Chi, Yijinjing, and CBT as primary interventions for managing depression. Their consistent effectiveness across multiple measures provides robust evidence for their integration into clinical practice and supports their role in evidence-based treatment strategies. Further details can be found in Figure 7.

### 3.5 Sensitivity analysis and publication bias

**Figure 8:**
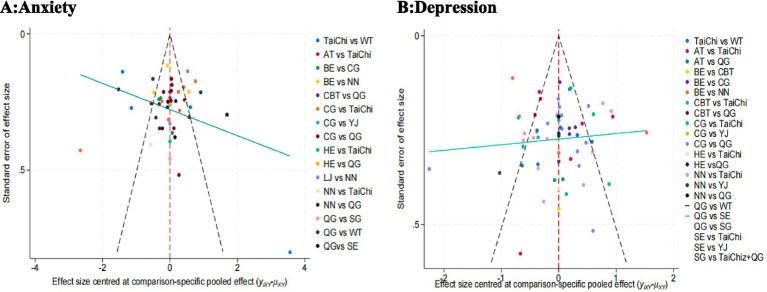
Publication bias testing. BE, Baduanjin; TC, Tai Chi; QG, Qigong; YJ, Yijinjing; SG, Sham Control; CBT, CBT; CG, Blank Control; HE. Home Exercise; NN, Standard Care; WT, Waiting Control; SE. Stretching; AT, Aerobic Training; LJ, Liu Zi Jue.

This study assessed publication bias using funnel plots. For anxiety, the funnel plot displayed slight asymmetry, suggesting a potential risk of publication bias, with uneven study distribution and the presence of some outliers. In contrast, the funnel plot for depression appeared more symmetric, indicating a lower likelihood of publication bias. Detailed results are shown in Figures 8A,B.

## 4 Discussion

The present systematic review and network meta-analysis provides a comprehensive evaluation of the comparative effectiveness of TCEs and other interventions in managing anxiety and depression among adults. By synthesizing evidence from a broad range of randomized controlled trials across diverse populations, this study offers valuable insights into the potential role of TCEs in mental health care.

Our findings underscore the substantial therapeutic benefits of TCEs, particularly Tai Chi, Liu Zi Jue, and Yijinjing, in alleviating symptoms of anxiety and depression. These results are consistent with previous meta-analyses that have demonstrated the positive effects of TCEs on mental health outcomes (13, 29). The mechanisms underlying these effects are likely multifaceted, involving a complex interplay of physical, psychological, and social factors. TCEs are characterized by gentle, low-impact movements, deep breathing techniques, and meditative components, which have been shown to promote relaxation, reduce stress, and enhance emotional regulation (30, 31). Moreover, the social support and sense of community that often accompany group-based TCEs practice may contribute to improvements in mood and overall well-being (32, 33).

Our analysis revealed that certain TCEs, such as Liu Zi Jue and Tai Chi, demonstrated promising efficacy in alleviating symptoms of anxiety and depression, showing encouraging results compared to established interventions like CBT in specific research contexts. These preliminary findings suggest that TCEs may serve as complementary therapeutic options, offering accessible, cost-effective, and culturally relevant alternatives for mental health interventions (17, 24). Given the global burden of mental health disorders and the limitations of current treatment approaches, such as high costs, limited accessibility, and potential side effects associated with pharmacological interventions (20, 34), the integration of TCEs into mental health care delivery could potentially expand access to effective treatments and improve patient outcomes, particularly in resource-limited settings or among populations with cultural preferences for non-pharmacological approaches. However, it is crucial to acknowledge the heterogeneity in intervention protocols, study populations, and outcome measures across the included trials. The inherent variability in TCEs practices and study designs may limit the generalizability of our findings (35, 36). To address this issue, future research should prioritize the standardization of intervention protocols, including the determination of optimal dosages, frequencies, and durations of practice. Additionally, investigating the long-term sustainability of treatment effects and the potential for maintenance programs to prevent relapse is essential for informing clinical guidelines and patient care (37, 38).

Another important consideration is the cultural context in which TCEs are practiced and studied. The majority of included trials were conducted in China, which may limit the external validity of our results to other cultural settings. However, this cultural context may actually be a contributing factor to TCEs’ remarkable effectiveness observed in our analysis. Research by Kwasnicka et al. suggests that behavioral changes might be produced and maintained more effectively when interventions are delivered within a culturally meaningful and acceptable framework (39). Their systematic review identified that interventions aligned with participants’ cultural identity and social context show improved adherence and outcomes, as individuals are more likely to maintain practices that resonate with their cultural values and beliefs (39) TCEs, deeply rooted in Chinese philosophical traditions and cultural practices, may facilitate better adherence and acceptance among participants who share this cultural background, potentially explaining the strong therapeutic effects observed in our study (40). As TCEs gain global popularity, it is imperative to examine their effectiveness and acceptability across diverse populations and healthcare systems (41). This requires collaborative efforts among researchers, practitioners, and policymakers to adapt and implement TCEs in a culturally-sensitive manner, while maintaining fidelity to core principles and practices (42). Engaging stakeholders, including patients, caregivers, and community leaders, in the design and implementation of TCE interventions can help ensure their relevance, feasibility, and sustainability in different cultural contexts.

Our study also highlights the need for further investigation into the differential effects of various TCEs on anxiety and depression. While Tai Chi, Liu Zi Jue, and Yijinjing demonstrated the most promising results, other practices such as Qigong and Baduanjin also showed potential benefits (43, 44). Future research should aim to elucidate the unique characteristics and mechanisms of action of each TCEs, as well as their suitability for specific subpopulations or clinical presentations. For instance, certain TCEs may be more effective for individuals with mild to moderate symptoms, while others may be better suited for those with severe or treatment-resistant disorders (45, 46). Additionally, exploring the potential synergistic effects of combining TCEs with other evidence-based interventions, such as psychotherapy or pharmacotherapy, could lead to the development of personalized, multimodal treatment approaches tailored to individual needs and preferences. Furthermore, the successful integration of TCEs into mental health care delivery will require addressing several key challenges and barriers. These include limited awareness and understanding of TCEs among healthcare providers, lack of standardized training programs for practitioners, and issues related to reimbursement and insurance coverage (12, 47). To overcome these obstacles, concerted efforts are needed to increase education and training opportunities for healthcare professionals, establish evidence-based guidelines and certification standards, and advocate for policy changes that support the integration of TCEs into mainstream healthcare systems (15, 48). Collaborations between researchers, clinicians, and policymakers can help bridge the gap between evidence and practice, facilitating the translation of research findings into real-world settings.

Moreover, as the field of mind–body interventions continues to evolve, it is essential to consider the potential role of technology in enhancing the accessibility, scalability, and personalization of TCEs. The development of digital platforms, such as mobile apps or virtual reality programs, could enable remote delivery of TCEs interventions, making them more widely available to individuals who may face barriers to in-person participation (49, 50). These technologies could also facilitate the collection of real-time data on patient adherence, progress, and outcomes, allowing for the optimization of treatment plans and the identification of early warning signs of relapse (51). However, the development and implementation of technology-based TCEs interventions must be guided by rigorous research to ensure their safety, efficacy, and user acceptability.

In conclusion, this systematic review and network meta-analysis provides strong evidence for the efficacy of TCEs, especially Tai Chi, Liu Zi Jue, and Yijinjing, in managing anxiety and depression in adults. To the best of our knowledge, this is the first meta-analysis using a network meta-analysis approach to determine the effectiveness of TCEs for anxiety and depression. The study evaluated and ranked the interventions, which can help clinicians and patients prioritize evidence-based interventions and make more informed decisions.

However, this study has several limitations that must be acknowledged. First, it should be noted that the article did not consider the intensity of the exercises analyzed, as most studies lacked this information. Second, due to the heterogeneity of the reviewed studies, the article could not provide recommendations on the suitability of each exercise based on the time after diagnosis and the patient’s recovery status. Third, in exercise-based interventions, it is difficult to blind participants and assessors, which further exacerbates the risk of bias. Fourth, the results should be considered with caution, as some interventions, including some of the most effective ones, had a limited number of studies. Fifth, due to the lack of available data, the research team was unable to determine the long-term effects of the interventions or the degree of adherence to the programs after the completion of the studies. Sixth, in comprehensive training, the interventions included in the studies were difficult to classify due to the different types of intervention combinations, which increased the heterogeneity of the results.

Despite these limitations, the findings highlight the potential of TCEs as complementary or alternative approaches to improving mental health outcomes. The integration of TCEs into mental health care delivery may offer a promising avenue for enhancing treatment options and improving the lives of individuals affected by anxiety and depression worldwide. This will require a multidisciplinary, collaborative approach that engages various stakeholders in the design, implementation, and evaluation of TCEs interventions, while also leveraging technological innovations to enhance their reach and impact. Future research should focus on establishing standardized protocols, determining optimal dosages, and evaluating the long-term effects and sustainability of TCEs interventions across diverse populations and cultural contexts.

## 5 Conclusion

This systematic review and network meta-analysis provides preliminary evidence for the efficacy of TCEs, particularly Tai Chi, Liu Zi Jue, and Yijinjing, in managing anxiety and depression in adults. Our findings suggest that these mind–body practices offer therapeutic benefits as potentially accessible, cost-effective, and culturally-relevant treatment options. These results require validation through more high-quality, multi-center, large-sample randomized controlled trials before direct application in clinical practice. Future research should focus on establishing optimal dosages, assessing long-term effects, and evaluating generalizability across diverse contexts. Nonetheless, this study highlights the potential of TCEs as promising complementary or alternative approaches to improving mental health outcomes in a field where expanded treatment options are needed.

## Data Availability

The original contributions presented in the study are included in the article/Supplementary material, further inquiries can be directed to the corresponding author.
